# Prediction of fracture load and stiffness of the proximal femur by CT-based specimen specific finite element analysis: cadaveric validation study

**DOI:** 10.1186/s12891-017-1898-1

**Published:** 2017-12-16

**Authors:** Michiaki Miura, Junichi Nakamura, Yusuke Matsuura, Yasushi Wako, Takane Suzuki, Shigeo Hagiwara, Sumihisa Orita, Kazuhide Inage, Yuya Kawarai, Masahiko Sugano, Kento Nawata, Seiji Ohtori

**Affiliations:** Department of Orthopedic Surgery, Graduate School of Medicine, Chiba University, 1-8-1 Inohana, Chuo-ku, Chiba city, Chiba, 260-8677 Japan

**Keywords:** Finite element analysis, Proximal femoral fracture, Validation study, Fresh frozen cadaver, Osteoporosis

## Abstract

**Background:**

Finite element analysis (FEA) of the proximal femur has been previously validated with large mesh size, but these were insufficient to simulate the model with small implants in recent studies. This study aimed to validate the proximal femoral computed tomography (CT)-based specimen-specific FEA model with smaller mesh size using fresh frozen cadavers.

**Methods:**

Twenty proximal femora from 10 cadavers (mean age, 87.1 years) were examined. CT was performed on all specimens with a calibration phantom. Nonlinear FEA prediction with stance configuration was performed using Mechanical Finder (mesh,1.5 mm tetrahedral elements; shell thickness, 0.2 mm; Poisson’s coefficient, 0.3), in comparison with mechanical testing. Force was applied at a fixed vertical displacement rate, and the magnitude of the applied load and displacement were continuously recorded. The fracture load and stiffness were calculated from force–displacement curve, and the correlation between mechanical testing and FEA prediction was examined.

**Results:**

A pilot study with one femur revealed that the equations proposed by Keller for vertebra were the most reproducible for calculating Young’s modulus and the yield stress of elements of the proximal femur. There was a good linear correlation between fracture loads of mechanical testing and FEA prediction (R^2^ = 0.6187) and between the stiffness of mechanical testing and FEA prediction (R^2^ = 0.5499). There was a good linear correlation between fracture load and stiffness (R^2^ = 0.6345) in mechanical testing and an excellent correlation between these (R^2^ = 0.9240) in FEA prediction.

**Conclusions:**

CT-based specimen-specific FEA model of the proximal femur with small element size was validated using fresh frozen cadavers. The equations proposed by Keller for vertebra were found to be the most reproducible for the proximal femur in elderly people.

## Background

Proximal femoral neck fracture is a major cause of high morbidity and mortality in elderly people with osteoporosis [1, 2]. A decline in the bone mineral density (BMD) is associated with fracture; hence, to predict fracture risk, dual energy x-ray absorptiometry and quantitative computed tomography (CT) have been widely used to measure BMD [3–7]. However, BMD is correlated only with bone strength and cannot indicate other mechanical properties of the proximal femur [8]. Furthermore, its ability to predict bone strength varies [9–11]. CT-based finite element analysis (FEA) can account for various aspects of the bone, such as bone geometry, cortical and trabecular bone distribution, and loading direction, and can improve the predictive accuracy of bone strength. The first validation study was conducted by Keyak et al. [12], and several studies have reported the accuracy and usefulness of CT-based FEA of the proximal femur in the stance configuration [13–17].

Recently, a large number of studies using FEA of the proximal femur have been reported as a postoperative evaluation with small implants like screws, plates, and the femoral stem [18–23]. In future, these studies may be useful for preoperative planning to select the most suitable implant or fixation methods. However, FEA for the bone behavior with implants is controversial because mesh sizes of models that were used in previous validation studies were relatively larger than implant sizes. Thus, FEA models should be validated with a smaller mesh size to improve their reliability.

This study aimed to document a proximal femoral FEA model using fresh frozen cadavers with smaller mesh size and to verify the accuracy of FEA prediction using CT-based specimen-specific FEA compared with mechanical testing.

## Methods

### Specimen

Twenty femora (right, 10 and left, 10) from 10 fresh frozen cadavers (males, 5 and females, 5) were obtained from the Clinical Anatomy Laboratory in our university. The mean age was 87.1 years [range, 74–101 years; standard deviation (SD), 9.13]. Subjects underwent no previous hip surgeries. The cause of death included pneumonia (*n* = 3); senility (*n* = 2); heart failure (*n* = 2); and chronic renal failure, breast cancer, and pancreatic cancer (*n* = 1 each). Cadavers were stored at −22 °C, and after thawing at room temperature, whole femurs were retrieved from the body and all soft tissues were removed. CT was performed using Aquilion ONE (Toshiba Medical Systems, Tokyo, Japan) with imaging parameters including 320-row detector; 120 kV; 200 mA; slice thickness, 0.5 mm; and pixel width, 0.3 mm. A calibration phantom (QRM-BDC, QRM, Möhrendorf, DE) containing three hydroxyapatite rods (0, 100, and 200 mg/cm^3^) was tested together with the specimen in water. The proximal femora were then sawed 12 cm distal from the tip of the greater trochanter, sloping 20° in the coronal plane to the shaft axis. Specimens were sprayed with a saline solution to maintain their moisture during the procedure and were not refrozen.

### Mechanical testing

**Fig. 1:**
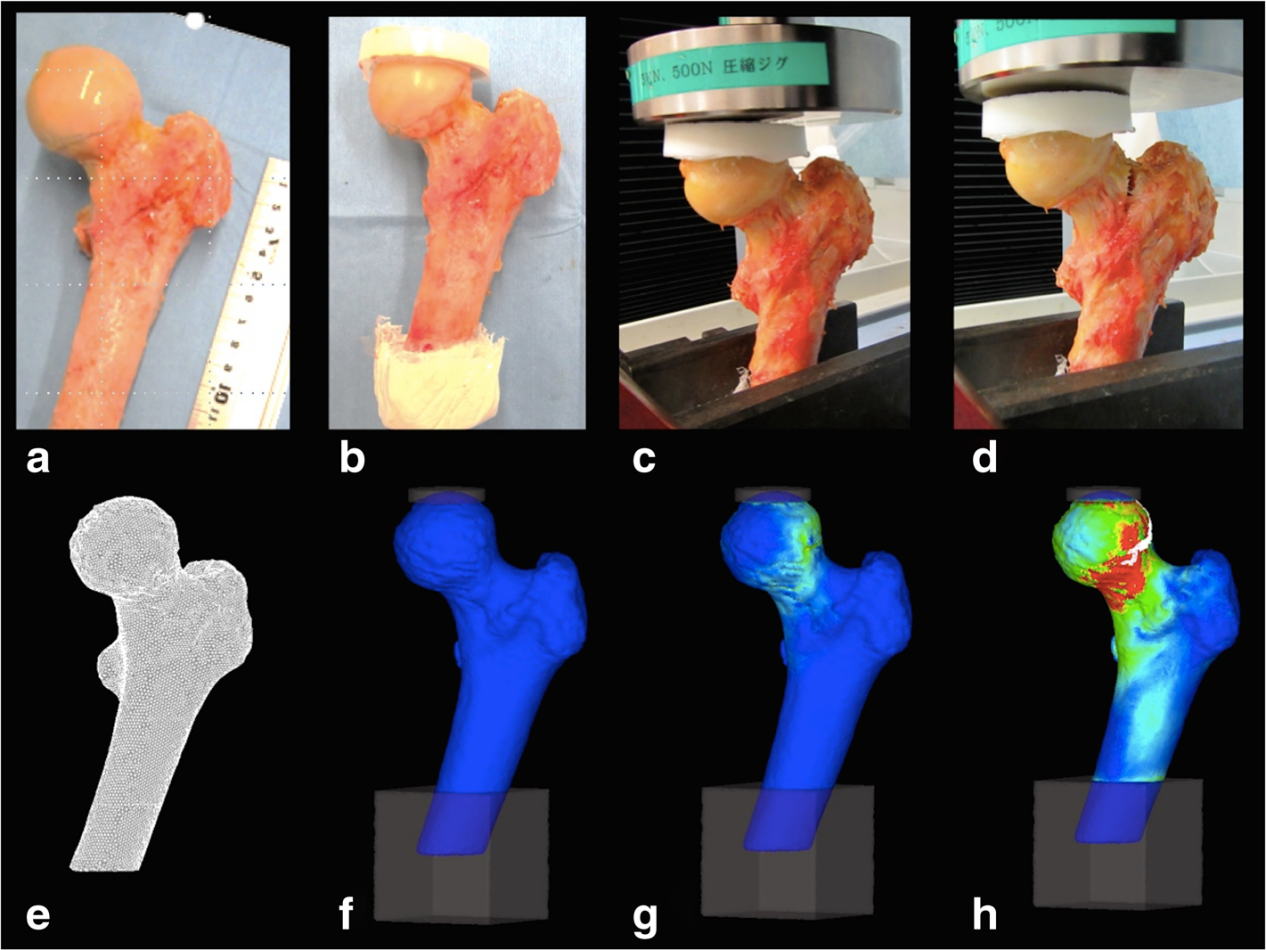
Process of mechanical testing and finite element analysis. A proximal femur is sawed 12 cm distal from the tip of the greater trochanter, sloping 20° in the coronal plane to the shaft axis (**a**). The distal 3 cm of the specimen is fixed using resin cement and a ‘resin cap’ is placed on the femoral head (**b**). The force is applied at a fixed vertical displacement (**c**) until the proximal femoral fracture occurs (**d**). The model of the proximal femur is made (**e**), is angled and fixed at the distal end with a resin cap on the femoral head (**f**). A compressive load was applied (**g**) until predicted fracture (**h**)

To verify fracture load assessment, quasi-static compression testing was conducted. Specimens were loaded using a universal testing machine (Autograph AG-20kN X Plus; Shimadzu, Kyoto, Japan). The distal 3-cm portion of each specimen parallel to the sawed surface was fixed using resin cement (resin box), and a resin cap was molded on the femoral head to apply a uniform compressive load (Fig. 1). The force was applied on the resin cap at a fixed vertical displacement rate of 5 mm/min until proximal femoral fracture occurred. The magnitude of the applied load and displacement were continuously recorded, and mechanical fracture was identified when the slope of the force–displacement curve (stiffness) rapidly decreased. Mechanical stiffness was calculated between 20% and 80% of the maximum fracture load using the force–displacement curve.

### Nonlinear FEA prediction

#### Model development

Data from CT were transferred to an HP Z400 workstation (Hewlett-Packard, Palo Alto, CA, USA). The proximal femur model (12 cm distal from the tip of the greater trochanter) was made using FEA software (Mechanical Finder, Research Center for Computational Mechanics, Tokyo, Japan). All femoral trabecular bone and inner parts of the cortex were meshed using linear tetrahedral elements with 1.5-mm global edge length and overlaid with 1.5 × 1.5 × 0.2-mm triangular shell elements simulating the outer cortex.

#### Material properties

CT value of each element was set as the average of the voxels contained in one element. Mechanical properties of each element were calculated in Hounsfield units (HU) [24]. There are several equations for calculating the Young’s modulus and yield stress for proximal femur. Therefore, we first performed a pilot study using CT DICOM data from an 85-year-old male to determine the most reproducible equations for proximal femur at this mesh size, and the most reproducible equations were adopted in subsequent tests. Modulus values of <0.01 MPa were designated as 0.01 MPa, and those >20 GPa as 20 GPa [25]. Young’s modulus and yield stress of the shell element were calculated, assuming its CT value was 1000 HU. Drucker–Prager equivalent criterion was adopted for the yield of the element [26]. The tensile yield stress was assumed to be 0.8 times the compressive yield stress, in agreement with previous studies [15, 27, 28]. Poisson’s coefficient for each element was set at 0.3 [29].

To reproduce the real mechanical testing, the FEA model was sloped at 20° in the coronal plane to the shaft axis and fixed with a resin box 3 cm distally, and a resin cap was placed on the femoral head. A uniaxial compressive load with a uniform distribution was applied on the resin cap. The degree of displacement and the reaction force of each point were recorded, and the force–displacement curve was determined. FEA-predicted fracture load was defined as the load when stiffness had declined by >20% of the estimated values, and FEA-predicted stiffness was calculated in the same way as for the actual mechanical test.

### Statistical analysis

Fracture load and stiffness were compared in mechanical testing and by FEA prediction using Student’s *t* tests and Pearson χ2 tests (SPSS 16.0, SPSS, Inc., Chicago, Illinois, USA). Age, gender, and side differences were calculated using multiple regression analysis as predictive factors for the fracture load in the proximal femur. A *p*-value of <0.05 was considered significant.

## Results

### Pilot study

**Fig. 2:**
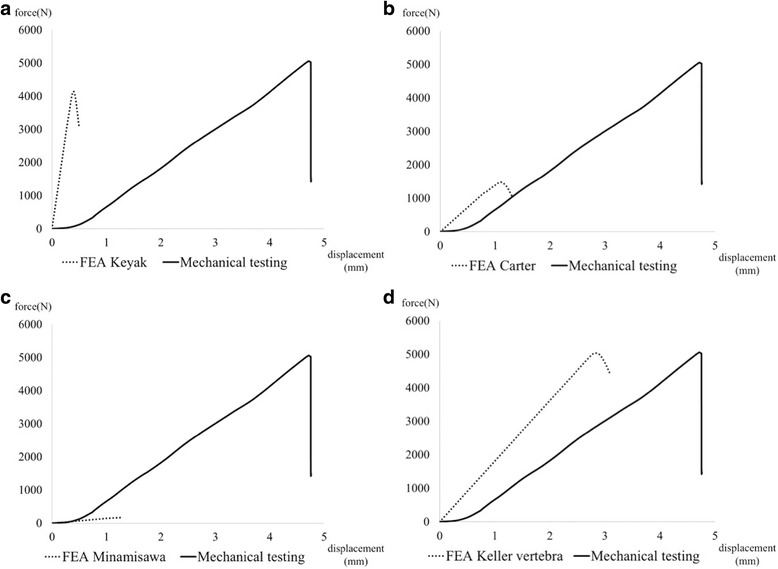
Preliminary force-displacement curves from mechanical testing and FEA prediction. The equations proposed by Keyak (**a**), Carter (**b**), Minamisawa (**c**), and Keller for vertebra (**d**). Predicted fracture load and stiffness were 0.82 times and 9.74 times (**a**), 0.29 times and 1.28 times (**b**), 0.16 times and 0.13 times (**c**), and 0.97 times and 1.57 times (**d**) larger than those from mechanical testing, respectively

The force–displacement curves obtained from mechanical testing and FEA prediction using equations proposed by Keyak [11] (Fig. 2a), Carter [30] (Fig. 2b), Minamisawa [31] (Fig. 2c), and Keller for vertebra [32] (Fig. 2d) are shown. Considering the fracture load (the tip of the curve) and stiffness (the slope of the curve), we concluded that Keller’s equation for vertebra was the most reproducible for the proximal femur. The following specific equations were used:

Young’s modulus (E, MPa).

$$ \mathrm{E}=0.001\ \left(\uprho =0\right). $$

$$ \mathrm{E}=1890\ {\uprho}^{1.92}\kern0.5em \left(\uprho <0\right). $$

Yield stress (σ, MPa).

$$ \upsigma =1.0\times {10}^{20}\kern0.5em \left(\uprho \le 0.2\right). $$

$$ \upsigma =284\ {\uprho}^{2.27}\kern0.5em \left(\uprho >0.2\right). $$

### Accuracy of FEA prediction in fracture load

**Fig. 3:**
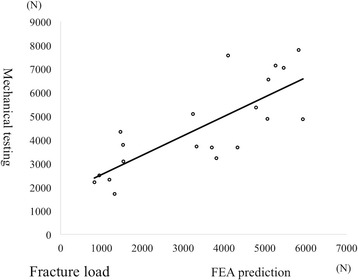
Relationship of fracture load between mechanical testing and the FEA prediction. (Mechanical fracture load) = 0.8201× (FEA predicted fracture load) + 1702.6, R^2^ = 0.6187, *p* = 0.001

The mean fracture loads from mechanical testing and FEA prediction using Keller’s equations for vertebra were 3435.8 N (SD, 1802.1) and 4520.3 N (SD, 1879.0), respectively. There was a good linear correlation between these values (Fig. 3).

### Accuracy of FEA prediction in stiffness

**Fig. 4:**
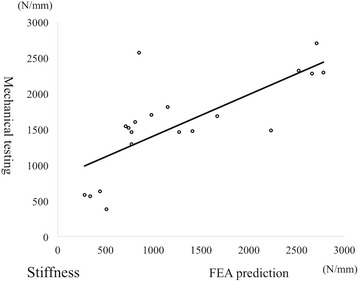
Relationship of stiffness between mechanical testing and the FEA prediction. (Mechanical stiffness) = 0.5810 × (FEA predicted stiffness) + 0.8223, R^2^ = 0.5499, *p* = 0.001

The mean stiffness from mechanical testing and FEA prediction were 1280 N/mm (SD, 847) and 1566 N/mm (SD, 664), respectively. There was also a good linear correlation between these values (Fig. 4).

### Correlation between fracture load and stiffness

**Fig. 5:**
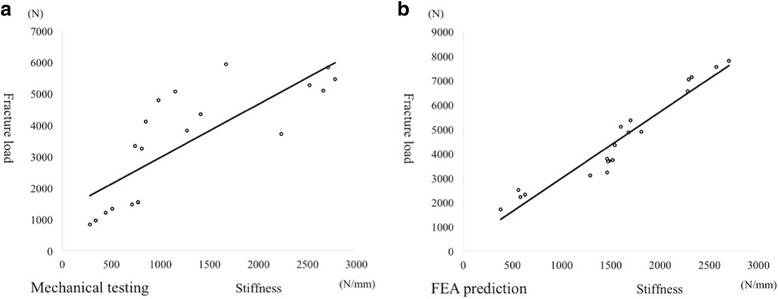
Relationship between fracture load and stiffness. Mechanical testing (**a**): (fracture load) = 1.6943 × (stiffness) + 1266.9, R^2^ = 0.6345, *p* = 0.001. FEA prediction (**b**): (fracture load) = 2.7207 × (stiffness) + 259.65, R^2^ = 0.9240, *p* = 0.001

There was a significant linear correlation between fracture load and stiffness in mechanical testing (Fig. 5a) and an excellent correlation between these in FEA prediction (Fig. 5b).

### Laterality in mechanical testing and FEA prediction

Fracture load and stiffness were not significantly different between the right and left femora for both mechanical testing and FEA prediction (Table 1).

**Table 1:** Laterality in mechanical testing and FEM prediction

	Right	Left	*p*
Mechanical testing			
Fracture load	3537.6 ± 1779.3	3333.9 ± 1914.9	0.762
Rigidity	1368.0 ± 880.5	1192.0 ± 850.2	0.706
FEM prediction			
Fracture load	4515.1 ± 2055.6	4525.5 ± 1796.5	0.940
Rigidity	1572.0 ± 712.1	1560.0 ± 650.5	0.821

Mean ± standard deviation

Mann-Whitney U test

### Effect of age on mechanical testing and FEA prediction

**Fig. 6:**
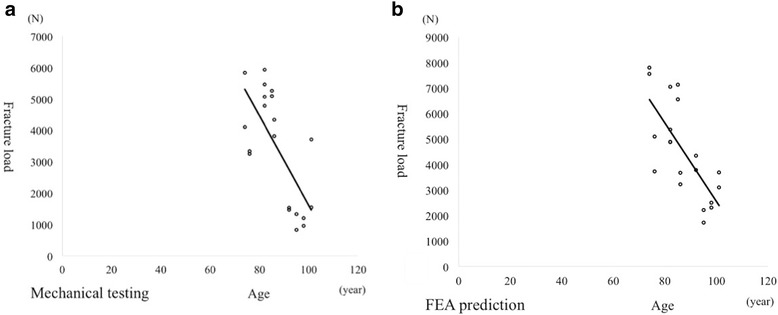
Relationship between fracture load and age. Mechanical testing (**a**): (fracture load) = −142.6 × (age) + 15,856, R^2^ = 0.4949, *p* = 0.001. FEA prediction (**b**): (fracture load) = −153.8 × (age) + 17,915, R^2^ = 0.5295, *p* = 0.001

There was a significant linear correlation between fracture load and age from mechanical testing; the load declined 142.6 N per year (Fig. 6a). FEA prediction also produced a significant linear correlation between fracture load and age, with load declining by 153.8 N per year (Fig. 6b).

## Discussion

FEA prediction was validated with a CT-based subject-specific proximal femoral FEA model with a smaller element size using fresh frozen cadavers. This study adopted a finer size tetrahedral element (1.5 mm) for the entire model than that reported in previous studies [13–17]. FEA models in previous studies used a 3.0-mm cube-shaped element [13], a l.5-mm cube-shaped element only below the lesser trochanter [14], a 3.0-mm tetrahedral element [15], or a 1.5–4.0-mm tetrahedral element [16, 17]. These element sizes are no longer sufficient to match the progress of modern implant technology and are insufficient to analyze the proximal femur with small screws or a stem-inserted model of the femur. Cube-shaped elements can make a mechanically precise model, but the model cannot be automatically made and is time-consuming to construct. An adequate model can be made using tetrahedral elements. At least 1.5-mm tetrahedral elements of the entire model are essential to simulate the proximal femur with small implants.

Preliminary testing revealed that Keller’s equations for vertebra were the most reproducible for calculating Young’s modulus and yield stress for the proximal femur. Although Keller’s equations for vertebra predicted approximately 1.6 times greater stiffness than mechanical testing, Keyak’s equations, which were adopted in several previous studies, predicted approximately 10 times greater stiffness than mechanical testing. We consider the error of Keller’s equations for vertebra to be acceptable and better than that reported in other studies. The correspondence between the proximal femur and vertebra derives from the high proportion of sponge-like cancellous bone with a thin cortical bone shell in both. Moreover, both proximal femur and vertebra present comparable risks for fracture in people with osteoporosis.

Correlations between mechanical testing and FEA prediction were significant for both fracture load and stiffness in this study. However, previous studies have shown extremely high correlations (fracture load, R^2^ = 0.73–0.96 and stiffness, R^2^ = 0.62–0.82) [13–17]. These discrepancies in the results may probably be due to differences in the equations adopted for FEA models. Smaller element size might be another reason for the discrepancy. It has been shown that differences in element size influence the results of FEA predictions [15, 33]. We adopted a compressive displacement condition to reproduce the results from mechanical testing, not to simulate the actual fracture situation, which may also have influenced the results. Furthermore, bone strength is determined not only by BMD but also by bone quality. Bone quality includes bone turnover, microarchitecture, collagen quality, mineralization, microdamage, and bone matrix and mineral composition [34–36]. Approximately 70% of bone strength is dependent on BMD and 30% on bone quality [37]. CT-based FEA can calculate the influence of BMD, but it does not consider all aspects of bone quality, which suggests that there is some systematic error inherent to FEA prediction. Nevertheless, we believe that our results are an acceptable estimate of mechanical behavior.

Good correlations between fracture load and stiffness have been reported previously [16]. This suggests that the fracture load of each specimen depends on its stiffness rather than on the degree of displacement at the point of fracture. The correlation between load and stiffness was far higher with FEA prediction than with mechanical testing. Bone quality, which was not considered in CT-based FEA, might have influenced these results.

Our study had certain limitations. First, most subjects were aged >80 years; hence, these results cannot be applied to younger people. However, FEA prediction of proximal femoral mechanical properties is most useful for elderly people at the risk of proximal femoral fractures. Second, we did not examine the fall configuration that was the most suitable to simulate actual hip fracture with a high correlation between mechanical and FEA-predicted values [12, 16, 38–40]. We only adopted the one-legged stance configuration to examine the behavior of the proximal femur because our focus was to validate the model and make it possible to analyze the model with small implants. Recent studies using FEA prediction of the proximal femur with small implants have provided much important knowledge [18–23]; however, the validation was insufficient. Third, we did not compare mechanical fracture sites with FEA-predicted fracture sites because most mechanical fracture sites were not obvious macroscopically or with CT imaging. The fracture sites could be evaluated with micro CT, but it was not practical or necessary for this study.

## Conclusion

FEA model of the proximal femur with small element size was validated using fresh frozen cadavers. The equations proposed by Keller for vertebra were found to be the most reproducible for the proximal femur in elderly people.

## Abbreviations

BMD: Bone mineral density
CT: Computed tomography
FEA: Finite element analysis
HU: Hounsfield units
